# Device-Detected Atrial Fibrillation: Why Time-Based Thresholds Are No Longer Fit for Purpose

**DOI:** 10.3390/jcm15082961

**Published:** 2026-04-14

**Authors:** Ahmed El-Medany

**Affiliations:** 1 National Heart and Lung Institute, Imperial College London, London SW3 6LY, UK; a.el-medany24@imperial.ac.uk; 2 Chelsea and Westminster NHS Foundation Trust, London SW10 9NH, UK; 3 Imperial College Healthcare NHS Trust, London W2 1NY, UK; 4 North Bristol NHS Trust, Bristol BS10 5NB, UK; 5 University Hospitals Bristol and Weston NHS Foundation Trust, Bristol BS1 3NU, UK

**Keywords:** atrial fibrillation, atrial high-rate episodes, stroke prevention, anticoagulation, artificial intelligence, atrial cardiomyopathy, wearable technology, cardiac implantable electronic devices, cardiac monitoring

## Abstract

Advances in implantable and wearable cardiac monitoring technologies have led to widespread detection of brief, often asymptomatic atrial high-rate episodes, frequently labelled as device-detected atrial fibrillation (AF). While detection has increased substantially, the clinical interpretation of these findings remains uncertain. Observational studies demonstrate associations between AF burden and stroke risk but reveal marked inter-individual heterogeneity and no consistent temporal threshold below which risk is eliminated. Recent randomised controlled trials show that anticoagulation guided solely by arrhythmia duration confers limited net clinical benefit, with modest reductions in ischaemic stroke offset by increased bleeding. These findings challenge the biological and clinical validity of rigid time-based thresholds for intervention. Increasing evidence suggests that AF may act primarily as a marker of underlying atrial disease rather than the sole mechanistic cause of thromboembolism. This article provides an evidence-informed perspective on the interpretation of device-detected AF in contemporary clinical practice and argues for a shift away from duration-based triggers toward a longitudinal, risk-adapted approach that integrates AF trajectory, atrial substrate, and clinical context. Emerging tools such as artificial intelligence-enhanced electrocardiography may help identify occult atrial pathology but must augment rather than replace clinical judgement. Proportionate, individualised care should supersede reflexive treatment strategies in the management of device-detected AF.

## 1. Detection Has Outpaced Interpretation

Continuous rhythm monitoring has fundamentally altered the landscape of atrial fibrillation (AF) detection. Cardiac implantable electronic devices (CIEDs), including pacemakers, defibrillators, and implantable loop recorders (ILRs), enable near-continuous rhythm surveillance in large patient populations. In parallel, consumer-grade wearable technologies capable of rhythm monitoring have expanded AF detection beyond traditional clinical settings, allowing scalable identification of previously undiagnosed arrhythmia in the general population [1,2].

As a result, brief and often asymptomatic atrial high-rate episodes (AHREs) are increasingly identified in individuals who would previously never have received an AF diagnosis. Observational studies have demonstrated that subclinical device-detected AF is common among patients with CIEDs and is associated with an increased risk of thromboembolic events, although the absolute event rates remain relatively low and vary considerably between individuals [3,4]. These findings have established device-detected AF and AHREs as clinically relevant entities while simultaneously highlighting the complexity of their interpretation.

The proliferation of wearable monitoring technologies has further accelerated this trend. Large population studies using smartwatch-based electrocardiography (ECG) and photoplethysmography have demonstrated that AF can be detected outside conventional healthcare settings with high sensitivity and scalability [1]. More recent investigations confirm that wearable monitoring may identify substantial numbers of previously undiagnosed AF cases, particularly among older individuals and those with cardiovascular risk factors [2]. While these devices offer important opportunities for earlier arrhythmia detection, they also raise new challenges regarding the interpretation of brief or isolated episodes.

This technological progress has created a paradox in contemporary arrhythmia care. While detection has become increasingly precise and sensitive, interpretation has not advanced at the same pace. Brief, device-detected AHREs now frequently prompt clinical discussions around anticoagulation, bleeding risk, and long-term stroke prevention, often in the absence of symptoms or other high-risk clinical features. For clinicians, this creates a recurring dilemma: whether to act early in the hope of prevention or to exercise restraint in the face of uncertain benefit. Recent reviews emphasise that although device-detected AF is associated with increased thromboembolic risk, the optimal management of short-duration episodes remains uncertain and continues to be debated [5,6,7].

For clinicians, this creates a recurring dilemma: whether to intervene early in the hope of preventing thromboembolism, or to exercise restraint when the absolute risk remains uncertain and treatment may expose patients to harm. In practice, time-based thresholds have emerged as pragmatic tools to navigate this uncertainty, with episode duration frequently used as a surrogate marker of thromboembolic risk. However, contemporary international guidelines increasingly acknowledge that the relationship between AF burden and stroke risk is complex and that rigid duration thresholds alone may not adequately guide therapeutic decision-making [8,9].

In the era of continuous monitoring, the central challenge is no longer whether AF can be detected but how its detection should meaningfully inform patient-centred care.

## 2. AF Burden and the Limits of Duration-Based Risk Stratification

Observational data initially supported the intuitive assumption that longer AF episodes confer greater stroke risk. The ASSERT study first demonstrated that device-detected subclinical AF was associated with an increased risk of thromboembolism in patients with implanted cardiac devices [3]. However, subsequent analyses revealed substantial heterogeneity in this relationship, with relatively low absolute event rates and considerable overlap in stroke risk among patients with differing AF burdens [4]. Further studies examining the temporal relationship between device-detected AF and thromboembolic events demonstrated that many strokes occur without a preceding arrhythmia episode, suggesting that AF duration alone may not fully capture underlying thromboembolic risk [10].

Systematic reviews and meta-analyses of device-detected AF cohorts have reinforced this interpretation. A dose-response meta-analysis of AF burden and stroke risk by Yang et al. (2022) demonstrated that increasing arrhythmia duration is associated with progressively higher thromboembolic risk, but without a clearly identifiable threshold below which risk is eliminated [11]. Meng et al. (2023) showed that AHREs detected by implanted devices report substantial variability in stroke risk among patients with comparable arrhythmia durations, highlighting the limitations of relying on episode length alone to guide treatment decisions [12]. Taken together, these findings suggest that AF burden reflects risk in a graded and probabilistic manner rather than through a discrete temporal threshold. Clinical risk scores such as CHA_2_DS_2_-VASc remain central to stroke risk stratification, with contemporary European guidelines adopting the CHA_2_DS_2_-VA score [8]. However, like AF burden, they provide probabilistic rather than deterministic estimates of risk, and do not directly capture the presence, severity, or progression of underlying atrial disease.

More recent randomised trials have provided critical insights into the consequences of treating device-detected AF on the basis of duration. The NOAH-AFNET 6 trial evaluated oral anticoagulation with edoxaban in patients with AHREs detected by implanted devices and reported a reduction in ischaemic events that was offset by increased major bleeding, resulting in no clear net clinical benefit [13]. Similarly, ARTESiA investigated anticoagulation with apixaban in patients with subclinical AF and demonstrated a reduction in stroke and systemic embolism accompanied by a significant increase in bleeding complications [14]. Importantly, these trials do not suggest that AF burden is irrelevant, but rather that duration alone is insufficient as a therapeutic trigger for anticoagulation.

Evidence from AF screening trials further underscores this limitation. In the LOOP study, systematic screening with ILRs substantially increased AF detection and initiation of anticoagulation but did not result in a significant reduction in stroke compared with usual care [15]. These data converge on a consistent message: increasing AF detection does not necessarily translate into improved outcomes, and burden-based treatment strategies risk exposing patients to harm without proportional benefit.

## 3. AF as a Marker of Atrial Disease

A growing body of evidence suggests that AF may function primarily as a marker of underlying atrial pathology rather than the sole mechanistic cause of thromboembolism. Structural remodelling, fibrosis, inflammation, endothelial dysfunction, and impaired atrial contractility may all contribute to stroke risk independently of arrhythmia duration, reflecting a broader disease process affecting the atrial myocardium [16,17]. This concept has been described as atrial cardiomyopathy, encompassing structural, architectural, contractile, or electrophysiological abnormalities of the atrial myocardium that may predispose to both AF and stroke [18,19,20].

Therefore, AF may represent a clinical manifestation of an underlying atrial disease process rather than the sole driver of thromboembolism. Studies examining markers of atrial cardiomyopathy, including left atrial enlargement, abnormal P-wave indices, and elevated natriuretic peptide levels, demonstrate associations with ischaemic stroke even in the absence of clinically documented AF [18,19,21]. These findings suggest that structural and functional abnormalities of the atrium may represent a shared substrate linking AF, atrial dysfunction, and thromboembolism.

**Figure 1:**
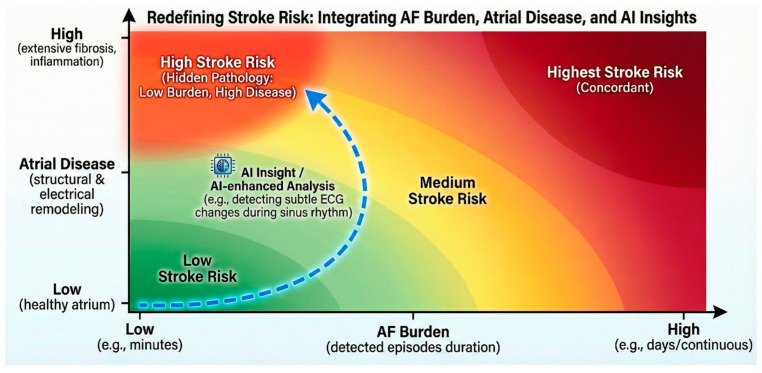
Conceptual relationship between atrial fibrillation (AF) burden, underlying atrial disease, and thromboembolic risk. Stroke risk is illustrated as a function of AF burden and underlying atrial disease or cardiomyopathy. The background gradient represents increasing thromboembolic risk, from low (green) to high (red). While a greater AF burden is traditionally associated with increased stroke risk, the upper left quadrant illustrates patients with brief AF episodes but advanced atrial disease who may carry substantial “hidden” risk. The dashed blue arrowed pathway indicates how artificial intelligence (AI)-based analysis (e.g., from sinus rhythm electrocardiograms) may identify occult atrial pathology, refining risk stratification beyond AF duration alone.

Within this paradigm, AF burden and atrial disease may therefore be better conceptualised as related but non-identical contributors to thromboembolic risk. AF episodes may reveal the presence of atrial pathology, but the severity and progression of that substrate may ultimately determine clinical outcomes. This conceptual relationship between AF burden, underlying atrial disease, and thromboembolic risk is illustrated in Figure 1. The clinically relevant question then shifts from “how long did the AF last?” to “what does the AF reveal about the atrial substrate?”

## 4. From Thresholds to Trajectories

Device-detected AF highlights the dynamic nature of arrhythmia progression. Rather than representing a static diagnosis, AF often evolves, with increasing episode frequency and duration reflecting progressive changes in the underlying atrial substrate. Longitudinal studies of device-monitored patients demonstrate that AF burden frequently increases over time and may be associated with structural and electrical remodelling of the atrium [5,17,22].

Within this context, the prognostic significance of device-detected AF may lie less in the duration of a single episode and more in the trajectory of arrhythmia burden over time. Progressive increases in AF burden, clustering of episodes, or transition from brief AHREs to sustained arrhythmia may reflect advancing atrial disease and rising thromboembolic risk.

Brief device-detected AF occurring in the context of advanced atrial disease may therefore warrant closer surveillance than longer episodes arising from relatively preserved atria (Figure 1). A trajectory-based approach reframes clinical restraint as active surveillance rather than inaction. Deferring anticoagulation after brief, isolated episodes acknowledges that risk is not fixed and allows intervention to be targeted when meaningful patterns of arrhythmia progression and atrial cardiomyopathy emerge, rather than when an arbitrary temporal threshold is crossed.

## 5. Artificial Intelligence and Risk Refinement

Artificial intelligence (AI) offers a potential means of addressing the limitations of time-based thresholds by shifting focus from arrhythmia duration to atrial substrate. AI-enhanced electrocardiography (AI-ECG) has demonstrated the ability to identify individuals at risk of developing AF while still in sinus rhythm, implying that atrial disease may be detectable before arrhythmia becomes clinically manifest [23]. Subsequent studies applying deep learning models to large ECG datasets have further shown that AI-based analysis can predict incident AF and identify patients with paroxysmal AF from sinus-rhythm recordings, highlighting the presence of subtle electrical signatures of atrial disease that are not apparent to human interpretation [24,25,26]. Notably, comparing conventional and AI-enhanced prediction strategies suggests that combining AI-ECG models with established clinical risk scores, such as CHARGE-AF, can achieve predictive performance comparable to more complex modelling approaches, reinforcing the potential role of AI as an adjunct, rather than replacement, to existing risk stratification frameworks [26,27]. More broadly, the expanding role of AI across the AF care pathway, from detection to risk stratification and therapeutic decision-making, has been comprehensively reviewed elsewhere, highlighting both its promise and current limitations [28,29].

Advances in digital health technologies have also expanded the scale and granularity of rhythm monitoring [1,2]. AI-ECG models originally developed using clinical ECGs are increasingly being adapted to analyse signals from wearable and single-lead ECG devices. For example, deep learning models applied to patch-based single-lead ECG recordings have been shown to predict near-term AF even in the absence of documented arrhythmia [30]. Other studies have demonstrated that single-lead ECG models derived from large 12-lead datasets can achieve predictive performance comparable to full 12-lead models, supporting the feasibility of translating AI-ECG risk prediction to wearable platforms [31]. Integrating AI algorithms with longitudinal ECG monitoring data may further enable individualised prediction of AF risk and disease trajectory, identifying patients most likely to develop clinically overt arrhythmia and therefore benefit most from stroke prophylaxis [32].

Beyond wearable technologies, similar approaches are increasingly being explored using data derived from CIEDs. Continuous rhythm monitoring from pacemakers, ILRs, and defibrillators generates large volumes of intracardiac electrogram (EGM) data that may be particularly suited to AI-based signal analysis. Machine learning models applied to intracardiac EGMs have demonstrated the ability to classify atrial arrhythmias and improve the accuracy of device-detected AF episodes, for example, by identifying characteristic electrogram signatures or reducing false-positive detections from ILRs [33,34,35].

Within the context of device-detected AF, these technologies may therefore help identify patients with advanced but clinically silent atrial pathology—those for whom even brief AF episodes may carry disproportionate thromboembolic risk. Conversely, they may also support reassurance in individuals with isolated or transient arrhythmia episodes occurring in otherwise healthy atria. In this sense, AI may help contextualise AF burden rather than amplify it.

However, these technologies must be applied cautiously. Algorithms trained on selective datasets may not generalise across diverse populations, and algorithmic predictions may be difficult to interpret when recommending lifelong therapy to asymptomatic individuals. Systematic reviews of AI-ECG prediction models highlight both the promise of these approaches and the need for prospective validation and careful evaluation of model generalisability [36]. AI-based tools should therefore augment clinical judgement rather than replace it and should be integrated within a broader longitudinal framework of risk assessment rather than used as binary triggers for intervention.

## 6. Implications for Practice

Translating advances in risk stratification into clinical decision-making remains challenging, particularly when improved detection does not clearly map onto improved outcomes. Even with emerging tools that promise finer discrimination of atrial disease, uncertainty persists at the point of care. Randomised trials evaluating anticoagulation in device-detected AF have demonstrated modest reductions in ischaemic events that are offset by increased bleeding, highlighting the difficulty of converting earlier detection into a clear net clinical benefit [13,14,15]. As a result, contemporary guidelines have increasingly shifted away from rigid, duration-based treatment triggers. Device-detected subclinical AF should therefore be interpreted not simply as an arrhythmia requiring treatment, but as a clinical signal of underlying atrial disease that justifies longitudinal evaluation.

Recent European and United Kingdom guidance does not endorse rigid duration-based thresholds for anticoagulation in device-detected AF, instead emphasising individualised decision-making and shared clinician–patient discussion [8,37]. This reflects an important shift away from reflexive treatment toward proportionate, risk-adapted care.

In practice, this means recognising that observation is not synonymous with neglect. Structured surveillance, reassessment of AF trajectory, and attention to evolving markers of atrial disease allow clinicians to intervene when risk becomes clearer. Equally important is transparent communication with patients, particularly around uncertainty, absolute risk, and the trade-offs between stroke prevention and bleeding. In an era of continuous monitoring, restraint, when appropriately justified, is itself an active clinical decision.

In practical terms, this framework encourages clinicians to interpret device-detected AF within a broader clinical context rather than responding reflexively to episode duration alone. For example, brief AHREs in a patient with significant structural heart disease, an elevated CHA_2_DS_2_-VA score, or imaging evidence of atrial remodelling may merit closer surveillance or earlier consideration of anticoagulation. Conversely, isolated short episodes detected in otherwise low-risk individuals may reasonably prompt continued monitoring rather than immediate treatment. Such an approach aligns with contemporary guideline recommendations and emphasises longitudinal assessment of AF burden, atrial substrate, and overall cardiovascular risk.

## 7. Conclusions

There is no universal temporal threshold that can substitute for clinical judgement in device-detected AF. AF duration matters only insofar as it reflects the presence, progression, and severity of underlying atrial disease. Treating AF minutes as interchangeable units of risk represents false precision rather than personalised care. The future of AF management lies in integrating longitudinal monitoring, substrate assessment, and emerging AI-based tools within a proportionate, patient-centred framework.

## Data Availability

No new data were created or analysed in this study. Data sharing is not applicable to this article.

## Abbreviations

The following abbreviations are used in this manuscript:

AI	Artificial Intelligence
AI-ECG	Artificial Intelligence-Enhanced Electrocardiogram
AF	Atrial Fibrillation
AHRE	Atrial High-Rate Episode
CIED	Cardiac Implantable Electronic Device
ECG	Electrocardiogram
EGM	Electrogram
ILR	Implantable Loop Recorder
